# Assessing the effectiveness of perimeter lockdowns as a response to epidemics at the urban scale

**DOI:** 10.1038/s41598-023-31614-8

**Published:** 2023-03-18

**Authors:** Alfonso de Miguel Arribas, Alberto Aleta, Yamir Moreno

**Affiliations:** 1 Institute for Biocomputation and Physics of Complex Systems (BIFI), University of Zaragoza, 50018 Zaragoza, Spain; 2 Department of Theoretical Physics, University of Zaragoza, 50018 Zaragoza, Spain; 3 Centai, Turin, Italy

**Keywords:** Computational models, Complex networks, Infectious diseases, Epidemiology

## Abstract

From September 2020 to May 2021 Madrid region (Spain) followed a rather unique non-pharmaceutical intervention (NPI) by establishing a strategy of perimeter lockdowns (PLs) that banned travels to and from areas satisfying certain epidemiological risk criteria. PLs were pursued to avoid harsher restrictions, but some studies have found that the particular implementation by Madrid authorities was rather ineffective. Based on Madrid’s case, we devise a general, minimal framework to investigate the PLs effectiveness by using a data-driven metapopulation epidemiological model of a city, and explore under which circumstances the PLs could be a good NPI. The model is informed with real mobility data from Madrid to contextualize its results, but it can be generalized elsewhere. The lowest lockdown activation threshold $\Theta$ considered (14-day cumulative incidence rate of 20 cases per every $10^5$ inhabitants) shows a prevalence reduction $>20\%$ with respect to the scenario $\Theta =10^3$, more akin to the case of Madrid, and assuming no further mitigation. Only the combination of $\Theta =20$ and mobility reduction $>90\%$ can avoid PLs for more than $>20\%$ of the system. The combination of low $\Theta$ and strong local transmissibility reduction is key to minimize the impact, but the latter is harder to achieve given that we assume a situation with highly mitigated transmission, resembling the one observed during the second wave of COVID-19 in Madrid. Thus, we conclude that a generalized lockdown is hard to avoid under any realistic setting if only this strategy is applied.

## Introduction

During the COVID-19 pandemic, we have become acquainted with a myriad of measures to halt or at least control the spreading of the novel coronavirus SARS-CoV-2 at different scales. Measures that are different from vaccination and medical treatments are called non-pharmaceutical interventions (NPIs). These may include travel bans, lockdowns of different geographical extensions, curfews, restrictions on occupancy in public closed spaces, or self-protection measures such as wearing masks or sanitation (see^1^ for a comprehensive review of NPIs within the context of COVID-19)^2–6^. The objective of most NPIs is to reduce contacts between individuals, with the ultimate goal of breaking transmission chains and, subsequently, controlling the spread and minimizing hospitalizations and deaths. However, certain NPIs may bring severe economic downturns^7–9^, dysfunctional supply chains^10–12^, and social backlash^13–15^. Therefore, competent authorities should be properly informed of the benefits and costs of the possible interventions, especially if these are enforced.

Given the negative impact that global lockdowns and quarantines inflict on societies, it is important to look for alternative strategies in successive COVID-19 waves. In this regard, a proposed alternative in the context of urban settlements is that of localized mobility restrictions, which is a more fine-grained restriction that acts only on the neighborhoods that are especially affected by an epidemic outbreak, instead of acting indiscriminately on the whole city. In Spain, the city of Madrid stands as a unique and paradigmatic example of pursuing the so-called perimeter lockdowns (PLs) at the level of Basic Health Zones (BHZ, subareas of the city defined by public health criteria). The main characteristic of these lockdowns was that they were implemented in highly integrated urban areas. As such, they restricted mobility in and out of the BHZ but allowed residents to go to work, attend academic activities, or for other essential purposes in the rest of the city, for which public transportation was available. Similarly, small businesses such as restaurants and shops were allowed to open, although at 50% maximum capacity. Hence, the lockdown was much more permeable than those implemented at larger scales, in which mobility is completely restricted between cities or regions. The case of Madrid reached the literature through a commentary article by members of Madrid’s regional public health counseling^16^. But the commentary received several responses that pointed to inaccuracies and lack of self-criticism given the severe impact of the epidemic in the region^17–20^. Two different studies approached the question through statistical analyses and concluded its lack of effectiveness at least in the real-life setting under consideration^21,22^. Independently, perimeter lockdowns within a large city were also implemented during the first wave of COVID-19 in Santiago de Chile. In^23^, the authors similarly conclude, through causal inference methods, that this kind of strategy is ineffective in the context of highly integrated human settlements, such as cities.

Due to the rarity of this strategy, to the best of our knowledge, it has not been explored using mechanistic models. In contrast to studies that rely on the statistical analysis of the collected data, mechanistic models can provide important insights into the effects of any strategy regardless of the peculiarities of the situation under analysis. This is important to properly gauge if a strategy will be effective in other places and under different conditions. Thus, in this work, we aim to assess the effectiveness of PLs at the urban scale using these models. To do so, we first take a brief look at the data collected during the implementation of the strategy in Madrid. This case serves as an example of how this strategy can be implemented in practice. Then, we build a simplified model of COVID-19 transmission to test the effect of PLs at the urban scale using a metapopulation framework. It is worth stressing that, even though we use the case of Madrid as an inspiration to conceive and guide the study, the framework developed is general enough to be extended to other locations. As such, we do not intend to replicate Madrid’s epidemiological trajectory but rather design, based on epidemiological first principles, a minimal model that captures the essential features of the spreading at the urban level and that can inform public health policy on the outcome of potential scenarios.

## Results

First, we take a brief look at the real data from Madrid during the period in which the PL strategy was in place. Then, we show the results from the simulations of our metapopulation model with PLs applied to an idealized scenario inspired by Madrid.

### Analysis of Madrid’s epidemiological data

**Figure 1:**
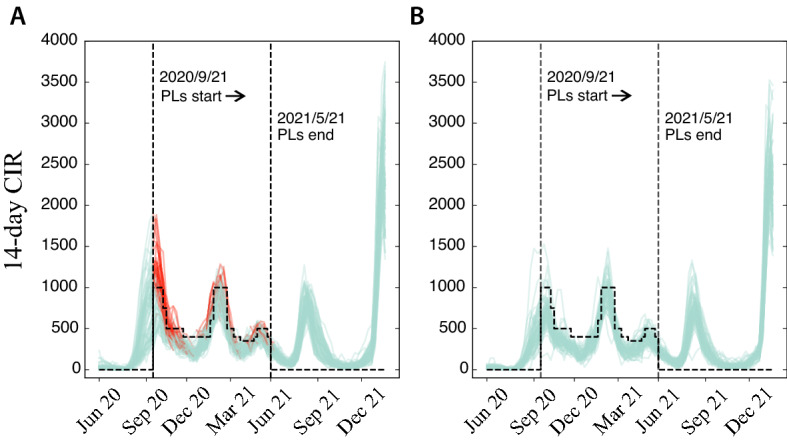
Madrid’s epidemiological trajectory. Real 14 day cumulative incidence rate time series for the basic health zones (BHZs) in Madrid city. (**A**) Trajectories for BHZs that during some time period experienced a perimeter lockdown (PL). In red, the period in which they were under a PL. (**B**) Trajectories for BHZs that were not confined (as extracted from the official bulletins). Vertical dashed lines mark the beginning and the end of the perimeter lockdown strategy in Madrid and step-wise horizontal dashed lines signal the risk threshold considered by the authorities to activate the lockdowns.

In general, perimeter lockdowns were enforced in areas in which the 14-day cumulative incidence rate (14d CIR) was above a certain threshold, although this information could be complemented by its trend and the, broadly defined, presence of community transmission in the area. In Fig. 1, the dashed horizontal lines indicate the threshold set by the authorities which, as we can see, was not static. Rather, it mostly followed the epidemic waves. This strategy was in place from late September 2020 to May 2021 (vertical dashed lines in the figure), a period that comprises the second and third waves of the epidemic in Spain (the first one is not shown in the figures). Given the typical time scale of the spreading, it has been shown that the change in trend after the implementation of the strategy cannot be attributed to it^21,22^. Note also that there were several BHZs with a 14d CIR above the threshold that were not confined (Fig. 1B). Lastly, we observe high synchronization in the spreading for all the BHZ time series, whether under lockdown or not. Thus, either the application of the strategy in a few areas had effects on the whole system, or it had limited consequences.

Next, we look at the overall mobility from the beginning of the pandemic to the end of the PL strategy, F.g. 2. The imposition of the national lockdown (first vertical red line) reduced mobility to 40% of the usual values. Then, it slowly increased following the progressive lifting of the restrictions during the summer of 2020, reaching mobility values of 70% of the baseline. The value is reduced once again to 50% of the baseline mobility during August 2020, which is the period in which, typically, people go on vacation in Spain. By the time the PL strategy was put in place, mobility was close to 70% on weekdays. In this period, we observe that the mobility levels tend to stabilize, except for the occasional public holidays, around that 70%. Averaging over the full period of PLs, mobility levels remain at 0.63 [0.62, 0.64], and, excluding weekends, increase to 0.67 [0.66, 0.68]. Thus, the impact of the PLs on the overall mobility within the city was negligible.

**Figure 2:**
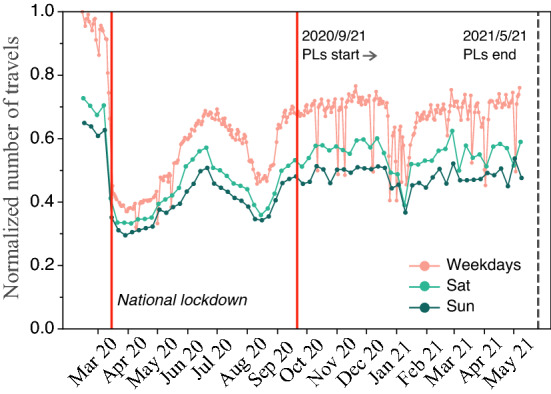
Madrid’s mobility patterns. Normalized total number of travels (with respect to the maximum value in the data) within the city of Madrid throughout the considered period. Weekend days are distinguished for the sake of clarity. There was a national lockdown from March 2020 to June 2020, corresponding to the first wave of the epidemic in Spain. The PL strategy was implemented in Madrid on late September 2020. Note that the Spanish Government stopped collecting mobility data two weeks before the end of the PL strategy in Madrid.

### Modeling perimeter lockdowns

We use a stochastic and discrete-time metapopulation SIR model (see “Methods”). In this context, a metapopulation is a collection of subpopulations that can exchange individuals. Inside each population, the dynamics of the disease are governed by the classical SIR model. Thus, an individual may get infected in one subpopulation, travel to another one and start a new outbreak there. These models have been applied to global-scale situations, such as the global spread of influenza-like diseases^24,25^, COVID-19^26–28^, and to regional and national scales^29,30^. The metapopulation framework^31,32^ is versatile and allows the description of a system on varying spatial scales, and thus can also be conceived on the urban scale. Our analysis considers an urban system with a total population of *N* individuals and *V* administrative subdivisions (subpopulations). The epidemic dynamics within each district *i* is governed by a well-mixed SIR compartmental model where the probability of getting infected is $1-[1-R_0\Delta t/(T_I N_i)]^{I_i}$, where $N_i$ represents the number of people in district *i*, $I_i$ is the number of infected individuals in the said area, $R_0$ is the basic reproduction number and $T_I$ is the mean infectious time.

The flow between districts in the metapopulation system is modeled through a static origin-destination matrix. The matrix was built using real mobility data from the pre-pandemic period (see “Methods”). We modulate the intensity of the flow with a global parameter, $\kappa$, so that if $\kappa \rightarrow 0$ individuals do not exit their district. Conversely, if $\kappa \rightarrow 1$, the mobility is equal to the baseline situation (see Eq. 2). We further assume a situation of general disease awareness in society and thus with generalized mitigation measures in place like wearing face masks, extra hygiene measures, some capacity limits, avoidance of crowded situations, etc. This awareness situation is more similar to the one experienced in Madrid at the time of the onset of the perimeter lockdown strategy. In this context, it would be more appropriate to model the spreading using the effective reproductive number $R_t$ rather than the basic reproductive number $R_0$, the latter being defined only for a fully susceptible population without any mitigation strategies in place. For simplicity, we set $R_0 \approx R_t$ to include directly all these factors without explicitly modeling them^33^. We set $R_0=1.25$ which is in the range of the estimations of the effective reproduction number performed by the Spanish Ministry of Health in August 2020, when the second wave of infections started^34^. Finally, we use an infectious period of $T_I=4.5$ days. All simulations are seeded with 5 infected individuals in the central district *Centro*. The simulations end when the total number of infected individuals in the system reaches zero.

We add two extra parameters to the model to reproduce the dynamics of the perimeter lockdowns (PLs): $\Theta$ and $\chi$. The first one is the risk threshold that determines when an area should be closed. Even though this parameter changed many times during the implementation of this strategy in Madrid (see Fig. 1), we fix it for each simulation. This way, whenever the cumulative incidence in a district *i* is above $\Theta$, a PL is activated in that area. As a consequence, flows of travelers from and to the district at risk are completely suppressed. The second parameter, $\chi$, is the transmissibility reduction fraction in district *i*, so that $\beta _i=\chi \beta$, where $\beta$ is the general transmissibility rate related to the basic reproductive number of the disease, $R_0=\beta T_I$. As previously discussed, PLs included other control measures aimed at reducing the spreading rate within the affected area, and this parameter allows us to control the intensity of such interventions.

To study the effect of PLs, we define three epidemiological observables: global peak incidence, $I_{\textrm{max}}$, global final prevalence, $R(\infty )$, and the final fraction of districts whose 14d CIR exceeded a certain risk threshold, $D(\infty )/V$. For brevity, we refer to these observables simply as the peak incidence, prevalence, and locked districts, respectively. Furthermore, in all cases the values are normalized over the ones obtained in the no-response scenario (subscripted as *nrs*), corresponding to a fully unmitigated outbreak. As such, when the normalized observables are less than unity, it signals that the intervention had some positive effect on containing the epidemic. In the following, we explore how these observables change when we vary the three free parameters in our model: the general mobility parameter, $\kappa$, the risk threshold, $\Theta$, and the transmissibility reduction, $\chi$.

### Effects of perimeter lockdowns

**Figure 3:**
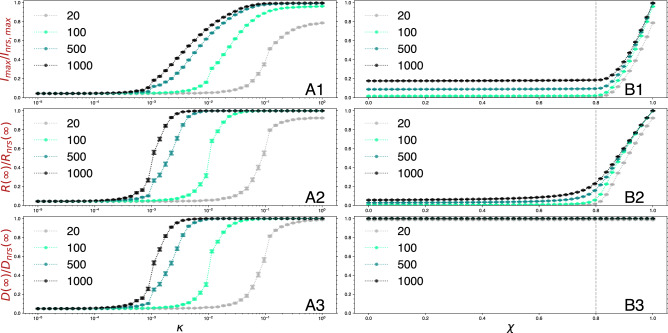
Epidemic impact curves for varying control parameter values. (**A1**), (**A2**), and (**A3**) show the peak incidence, prevalence, and locked districts fraction, respectively, for varying values of mobility parameter $\kappa$ and different threshold $\Theta$ values, without transmissibility reductions in the locked areas ($\chi =1$). (**B1**), (**B2**), and (**B3**) show the same observables for varying values of transmissibility reduction, $\chi$, and different $\Theta$ values, with baseline mobility ($\kappa =1$). Quantity values are normalized with respect to a no-response scenario. The vertical dashed line in the B panels marks the point where, by reducing $\chi$, the global reproduction number turns $R^*=1$, and thus the threshold under which the spread is under control^31^.

Panels A1 (peak incidence), A2 (prevalence), and A3 (locked districts) of Fig. 3 show a series of curves for several threshold risk values $\Theta =20$, 100, 500, and 1000, without transmissibility reduction in locked areas ($\chi =1$) and varying $\kappa$ as control parameter. Every point in the curves depicts a situation where mobility is lower than the baseline scenario ($\kappa =1$). Note that, since $\chi =1$, the only effect of PLs is to cut down mobility in and out the affected districts. These curves then allow us to disentangle the effects of general mobility on the system. Clearly, all the observables show that reducing the general mobility parameter $\kappa$ helps to reduce the impact of the epidemic with respect to the no-response scenario. The problem, though, lies in the magnitude of the decrease. Madrid’s PLs pursued risk threshold values mainly between $\Theta =500$ and 1000 most of the time. Under these conditions, even with lower mobility than the one observed during the national lockdown ($\kappa \sim 0.4$, see Fig. 2) the effects are mostly negligible. To achieve, for instance, a reduction of $20\%$ in peak incidence, mobility should be one order of magnitude lower than during the national lockdown ($\kappa \sim 0.01$). And this still would not impact the prevalence or the fraction of locked districts. Only when $\kappa =10^{-3}$ ($99.9\%$ reduction), these quantities are reduced by $50\%$. With a much more strict threshold for PL activation, $\Theta =20$, and under the regular mobility scenario, we can obtain a reduction in peak incidence $>20\%$, and around $10\%$ in prevalence, but this does not keep the system from undergoing a *de facto* general lockdown unless mobility is also reduced by $90\%$ or more.

In panels B1 (peak incidence), B2 (prevalence), B2 (locked districts) of Fig. 3, instead, we fix the general mobility to the baseline, $\kappa = 1$, and vary $\chi$ in the districts under PLs. In this situation, the intervention restricts the in and out mobility in the selected districts and also reduces the transmissibility within the area by a factor $\chi$. Here we readily see that PLs, when accompanied with additional mitigation measures inside the affected area, can effectively reduce the impact of the disease on the system. Given the proposed $R_0=1.25$ and $T_I=4.5$ days, reducing baseline transmissibility by $20\%$ ($\chi =0.8$) translates into an effective reproduction number for a quarantined district that is on the verge of criticality, that is, $R^*=1$^31^. Note, however, that the value of $R_0$ is already very low as we assume that multiple non-pharmaceutical interventions are in place. Thus, achieving further reductions might not be possible. In any case, when $\chi =0.8$ and $\Theta =500$, we obtain normalized values for peak incidence of 0.093 [0.093, 0.093] and prevalence of 0.166 [0.166, 0.167]. This is a reduction of more than $90\%$ and $80\%$ with respect to the no-response scenario, respectively. However, we can see that all districts in the urban system have outbreaks above the risk threshold, for any $\Theta$ and $\chi$ shown in the figure. Thus, under these conditions, perimeter lockdowns can greatly reduce the impact on the population but would not protect parts of the city from the most affected ones, even under mild epidemiological conditions.

**Figure 4:**
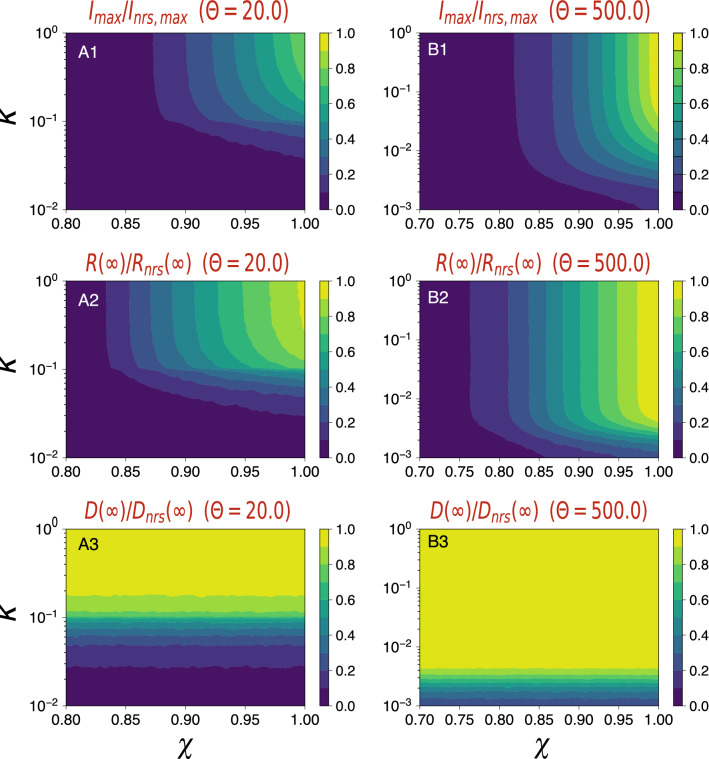
Epidemic impact when varying simultaneously $\kappa$ and $\chi$ for selected $\Theta$ scenarios. Plots (**A1**), (**A2**), and (**A3**) show the peak incidence, prevalence, and locked districts fraction, respectively, in $(\chi ,\kappa )$-space for the threshold $\Theta =20$. Plots (**B1**), (**B2**), and (**B3**) show the same observables for $\Theta =500$. All values are normalized with respect to a no-response scenario.

Lastly, we explore in more detail the space of parameters $(\kappa ,\chi )$ for two selected $\Theta$ scenarios. We consider a rather proactive strategy, with $\Theta =20$ (panels A1, A2, A3 in Fig. 4), and a more reactive strategy, with $\Theta =500$ (panels B1, B2, B3 in Fig. 4). With this analysis, we can better appreciate the limited role of general mobility $\kappa$ in both the reduction of the peak incidence and the final global prevalence when $\kappa$ decreases from unity down to sensible but really strict values (around $\kappa =0.1$). Now, moving from $\chi =1$ to $\chi =0.8$ we can obtain an important reduction of the impact, both for $\Theta =20$ and also $\Theta =500$. See Tables 1 and 2 for an outline of the results given a selection of $(\kappa ,\chi )$ scenarios. Finally, with respect to locked districts, with $\Theta =20$ it is possible to avoid the invasion of the entire system, but it requires a strong reduction of mobility, going well below $\kappa =0.5$ (see Table 1). Note the difficulty of doing this: PLs have to be activated very soon and, at the same time, it is essential to achieve a strong reduction of global mobility in the system to effectively save a few districts from experiencing an outbreak. With $\Theta =500$, the full invasion is unavoidable, except for extremely low values of mobility.

**Table 1:** Epidemic impact with the threshold $\Theta =20$.

	$$\Theta =20$$			
	$\chi =0.8$	$\chi =0.9$	$\chi =1$	
$\kappa =1$	0.01 [0.01, 0.01]	0.43 [0.42, 0.43]	1.69 [1.68, 1.70]	$I_{\textrm{max}}\%$
$\kappa =0.5$	0.01 [0.01, 0.01]	0.42 [0.42, 0.43]	1.63 [1.62, 1.64]	
$\kappa =0.1$	0.01 [0.01, 0.01]	0.31 [0.30, 0.32]	0.97 [0.94, 1.00]	
$\kappa =1$	0.67 [0.65, 0.68]	17.16 [17.05, 17.28]	34.24 [34.12, 34.37]	$R(\infty )\%$
$\kappa =0.5$	0.65 [0.63, 0.66]	17.13 [17.02, 17.24]	34.07 [33.92, 34.22]	
$\kappa =0.1$	0.52 [0.51, 0.54]	13.80 [13.40, 14.20]	27.04 [26.35, 27.74]	
$\kappa =1$	98.72 [98.57, 98.87]	98.72 [98.57, 98.88]	98.80 [98.63, 98.96]	$D(\infty )\%$
$\kappa =0.5$	97.33 [97.08, 97.57]	97.44 [97.22, 97.67]	97.19 [96.89, 97.49]	
$\kappa =0.1$	71.20 [69.92, 72.47]	75.71 [73.82, 77.61]	74.44 [72.59, 76.29]	

Average values as a percentage of the total population *N* for $I_{\textrm{max}}$ and $R(\infty )$, and as a percentage of system size *V* for locked districts $D(\infty )$, together with a 95$\%$ confidence interval in brackets for selected scenarios of $(\kappa ,\chi )$ values under $\Theta =20$. NRS gives $I_{\textrm{max}}=2.15$ [2.15,2.15], $R(\infty )=37.14$ [37.12, 37.16] and full invasion.

**Table 2:** Epidemic impact with the threshold $\Theta =500$.

	$$\Theta =500$$			
	$\chi =0.8$	$\chi =0.9$	$\chi =1$	
$\kappa =1$	0.20 [0.20, 0.20]	0.73 [0.73, 0.73]	2.13 [2.13, 2.14]	$I_{\textrm{max}}\%$
$\kappa =0.5$	0.20 [0.20, 0.20]	0.73 [0.73, 0.73]	2.13 [2.13, 2.14]	
$\kappa =0.1$	0.20 [0.20, 0.20]	0.73 [0.73, 0.73]	2.11 [2.11, 2.11]	
$\kappa =1$	6.19 [6.17, 6.20]	21.98 [21.97, 22.00]	37.13 [37.11, 37.15]	$R(\infty )\%$
$\kappa =0.5$	6.19 [6.17, 6.20]	21.98 [21.96, 21.99]	37.13 [37.12, 37.14]	
$\kappa =0.1$	6.17 [6.16, 6.19]	21.99 [21.97, 22.00]	37.12 [37.11, 37.14]	
$\kappa =1$	100 [100, 100]	100 [100, 100]	100 [100, 100]	$D(\infty )\%$
$\kappa =0.5$	100 [100, 100]	100 [100, 100]	100 [100, 100]	
$\kappa =0.1$	100[100, 100]	100 [100, 100]	100 [100, 100]	

Average values as a percentage of the total population *N* for $I_{\textrm{max}}$ and $R(\infty )$, and as a percentage of the size of the system *V* for the locked districts $D(\infty )$, together with 95$\%$ confidence interval in brackets for selected scenarios of values of $(\kappa ,\chi )$ under $\Theta =500$. NRS gives $I_{\textrm{max}}=2.15$ [2.15,2.15], $R(\infty )=37.14$ [37.12, 37.16] and full invasion.

## Discussion

Examples of perimeter lockdowns of highly dense-integrated suburban areas are scarce. The inspection of the real epidemiological and mobility data from Madrid casts a lot of doubts about its proper implementation and its effectiveness. As we referenced previously, this is something that the few works devoted to the experience of Madrid have formally confirmed^21,22^. In^21^, through a joint point trend analysis, the authors found that the decrease in the epidemic curve, both in the entire city and the BHZ affected, started before the impact of the perimeter lockdown could have been reflected, and also found that the strategy did not increase the speed at which cases were decreasing. The authors offer several reasons for the strategy’s failure: (1) the curve started to decrease before measures were taken. (2) Perimeter lockdowns were focused on mobility rather than preventing high-risk situations. (3) Even though the focus lied on mobility, it was allowed for essential activities such as working. And (4), BHZ boundaries were unknown to many residents since these are used for public healthcare administration. Regarding our particular experiment, points (2) and (3) apply and align well with our conclusions: mobility is not very much effective in this kind of setting, and the focus should be on preventing high-risk situations like workplace or community mixing. In^22^, through the use of a generalized additive models technique, they reach a similar conclusion: the perimeter confinements did not have a significant impact on the 14-day cumulative incidence.

To further understand the mechanisms underpinning the spreading dynamics in this strategy, we conceived a minimal model using a metapopulation framework parameterized using real mobility data. Our model results show how difficult it is to control an outbreak in an urban environment under the PLs. The reduction is critically marked by how successful the transmissibility reduction is within a district, with mobility playing a minor role. Note that Madrid initially set its lockdown risk threshold at $\Theta =1000$ and then changed it dynamically up and down, being $\Theta =350$ the lowest documented. In our model, even when the threshold was $\Theta =20$, a very high effort on reducing general mobility was needed to avoid invasion of the full system. This mobility reduction was not even achieved during the national lockdown that closed most businesses and industries (Fig. 2). Furthermore, during a PL in Madrid, it was still allowed to go to your workplace even if it was in another district.

Localized lockdowns were also implemented in Santiago de Chile during its first COVID-19 wave. In^23^, Li et al. explored their performance, obtaining mixed results. The authors state that localized lockdowns on their own are insufficient to control pandemic growth in the presence of indirect effects from contiguous neighboring areas that do not have lockdowns. As we did, they found that localized lockdowns can help contain the transmission of the virus but their effectiveness is dependent on its duration and indirect effects from neighboring areas with high social interconnectivity. Moreover, their estimates showed that in Greater Santiago the epidemic is only controlled when generalized lockdowns are in place. In contrast, these measures showed promising results in municipalities that were rather isolated from affected neighboring areas. They concluded that the growth of disease transmission is reversed only when lockdowns are implemented in a coordinated fashion across interconnected geographic areas.

Urban systems for the most part are typically very well interconnected systems where it is easy to move from one part to another in time scales significantly lower than 1 day. Large portions of the population in these systems mix at different sites during the day: whether at workplaces, at schools, at the public transportation system, or during leisure activities. Therefore, a high level of synchronization in the spreading can be expected. Hence, when an outbreak emerges in one area, it will quickly spread through the system unless mobility is extremely reduced. If perimeter lockdowns are accompanied by significant transmissibility reductions, the measures can make a difference to flatten the curves. But unless the action is performed soon enough, the most probable course of action for the spreading is to quickly invade the full system. If the goal of the PLs is to protect some parts of the system so that citizens inside can normally live and perform their daily activities, this seems unachievable under reasonable assumptions and regular behavior. Our results from a sound theoretical model which idealized several critical features and thus, overall, offered a best-case scenario, seem to confirm this thesis. Moreover, the real epidemiological data for Madrid clearly shows the synchronization phenomena among different areas. Responses that aim to partially isolate areas of a system that are so spatially interconnected and temporally synchronized are likely to be insufficient. As results have shown, the emphasis should not be on mobility (either general or among particular areas) but on general measures that bring transmission down where the mixing or contact is effectively produced.

Our model explains the qualitative features of the real epidemic spreading observed in Madrid under the PLs and sets a general model to approach this type of situation. Nonetheless, it has its limitations. As we have already stated, our model is minimal and is intended to capture only the most basic features of the spreading together with the control strategy, but it is not intended to reproduce the real COVID-19 trajectories that took place during the period studied. COVID-19 is a complex disease with a long latency period, an important amount of pre-symptomatic infectivity and even asymptomatic transmission. Moreover, the disease can present high hospitalization and death rates which are highly age-dependent. These are elements worth taking into account when aiming for accurate COVID-19 forecasting. However, regarding the assessment of non-pharmaceutical interventions as the one presented here, the SIR model stands as a simple but best-case scenario modeling choice. The rationale is that even though we neglect key elements that would hinder surveillance and disease control, such as a latency period and asymptomatic disease progression, the outcome is still unfavorable for the success of the strategy. Another limitation is that, at the subpopulation level, a homogeneous well-mixing scheme is considered. This is a standard assumption within the metapopulation framework but indeed relevant heterogeneous features may exist both intra-district and inter-district, such as age differential mixing. We also remark that our implementation of PLs assumed perfect information and absolute compliance. Moreover, travel bans were totally cut down, which is at odds with the permeability allowed in the real-life setting. Thus, even though our model is limited in many aspects, it can be, in general, regarded as a best-case scenario. The fact that the PLs did not achieve high effectiveness under these circumstances signals that in a real setting the situation would be worse.

Even though our minimal model indicates that the effectiveness of perimeter lockdowns at the urban scale is low, more detailed modeling could unveil subtleties at lower scales. Future works could aim for increased heterogeneity in human behavior. One could use micro-mobility models based on points of interests at the subpopulation level to break the homogeneous mixing assumption. In terms of the population, the addition of the socioeconomic characteristics of the population may help to disentangle the differential impact of non-pharmaceutical strategies on the diverse population strata. Indeed, even though at the aggregated level perimeter lockdowns appear to be inefficient, their impact on the most vulnerable parts of the population is yet unknown and is open for further research. From a theoretical point of view, an interesting research line would be to determine the amount of spatial cohesion at which these systems tend to be highly synchronized and for which mobility plays only a minor role beyond the initial stages. This could facilitate the analysis of strategies similar to the one presented here at multiple scales, such as metropolitan areas or urban-rural systems.

## Methods

### Madrid’s surveillance data

To test the effectiveness of the measures in the particular case of Madrid, we have explored the official epidemiological data reported by the government^35^ and collected the time series for the 14-day cumulative incidence rate (14d CIR) for all the BHZs belonging to the city of Madrid. The 14d CIR is a standard in the field of public health^36^ and is measured as:

1 $$\begin{aligned} \textrm{14d}\;\textrm{CIR}=\frac{\sum _{i=1}^{14}\textrm{new}\;\textrm{cases}_i}{N}\times 10^5 \;\textrm{inhabitants}, \end{aligned}$$

where *N* is the total population assuming a fully susceptible population at the beginning of the outbreak, and the summation of new cases is done over the previous 14 days. The value is given for every 100, 000 inhabitants. Therefore, for the sake of brevity, all references to this variable will omit this factor.

In Madrid, this indicator was used to determine whether or not a particular BHZ had to be confined, together with other auxiliary information such as its trend or the presence of community transmission. The threshold value upon which a PL was implemented varied in time. Consequently, for a more complete picture, we also reconstructed the 14d CIR threshold, or activation threshold, by looking at official bulletins^37^. The confined status of a BHZ was held for two weeks and then reviewed according to its epidemiological situation.

### Theoretical framework: Metapopulation model

The metapopulation framework is a versatile way of modeling the spreading of an epidemic in which the spatial distribution of individuals plays a major role^31,32^. In particular, we create a metapopulation system composed of 21 subpopulations representing each of the 21 administrative districts of the city of Madrid, Spain. Within each subpopulation, the epidemic dynamics are governed by the classical SIR model under the well-mixed approximation. It must be noted that, even though this model cannot capture some of the particular characteristics of COVID-19, it can still provide useful insights for settings in which those characteristics do not play a major role, as in our analysis^33,38^. In cases in which case detection or the latency period might be relevant, it would be necessary to use an SEIR model or one of its variants.

#### Epidemic modeling

At the district level, we implement a well-mixed SIR compartmental model. In this model, individuals are classified according to their health status: susceptible (S) if they are susceptible to catching the disease, infected (I) if they have been infected and can infect others, and removed (R) when they are either recovered or deceased. Within each district, the transition between compartments results from the following rules, iterated at each time step, corresponding to $\Delta t=1$ day^39^:

- $S \rightarrow I$: Susceptible individuals in district *i* are infected with probability $P_i(S\rightarrow I)=1-[1-R_0\Delta t/(T_IN_i)]^{I_i}$, where $R_0$ is the basic reproduction number of the disease, $T_I$ is the mean infectious time, $N_i$ represents the number of people in district *i* and $I_i$ represents the number of infected individuals in the said area.
- $I \rightarrow R$: Infected individuals become removed at a rate inversely proportional to the mean infectious period, $T_I$. The probability for an infected individual to transition to the removed state is thus simply $P(I\rightarrow R)=\Delta t/T_I$.

The new infected and removed individuals are generated stochastically by sampling from binomial distributions using the above probabilities, respectively^39,40^.

#### Mobility

To estimate realistic mobility patterns we use the data from a mobility survey carried out by the Spanish Ministry of Transport, Mobility and Urban Agenda^41^. This survey provides the estimated number of individuals going from one district to another every day since February 2020 to May 2021. We use the data from the pre-pandemic period to build a baseline mobility matrix that is not influenced by any changes on social interactions produced by the pandemic. Indeed, as described in the main text, we use an effective value of the basic reproduction number that already incorporates all these effects. Thus, if we were to use the real data from the period under study, we would be counting twice certain interventions.

Each element of the mobility matrix, $D_{ij}$, represents the rate at which individuals move from district *i* to district *j*. This can be estimated from the origin-destination (OD) matrix provided by the Spanish government, *M*, in which $M_{ij}$ represents the number of individuals traveling from district *i* to district *j*. In particular, the elements of *D* are defined as:

2 $$\begin{aligned} D_{ij}={\left\{ \begin{array}{ll} \kappa \frac{M_{ij}}{\sum _j M_{ij}} \quad &{}\text {if} \, i\ne j \\ 1-\sum _{k\ne i} D_{ik} \quad &{}\text {if} \, i=j \\ \end{array}\right. } \end{aligned}$$

where $D_{ij}$ is the rate at which individuals move from district *i* to district *j* and $\kappa$ is a general mobility parameter. The baseline values of $D_{ij}$ are obtained considering $\kappa =1$. When $\kappa \rightarrow 0$, we see that $D_{ii}\rightarrow 1$, so that the rate of individuals staying at their origin district approaches one. The particular values of $D_{ij}$ are calculated using data from the Ministry’s survey from the pre-pandemic period. Then, at each step $\Delta t$, the number of travelers from district *i* to every destination *j* is sampled from a multinomial process with probabilities $D_{ij} \Delta t$.

#### Perimeter lockdown strategy

The response against the spreading process is assumed to be the best-case scenario, with perfect information and neither exceptions nor violations of the policies enacted by the authorities. The observable to monitor the effectiveness in every district is the same as in the real-life setting, the 14d CIR. We set a risk threshold $\Theta$ so that whenever $14\textrm{d}\;\textrm{CIR}_i\ge \Theta$ for district *i*, we say that this district is at risk and thus it is set under a perimeter lockdown. The implications for a district under this type of lockdown are twofold:

1. Travel bans. Flows of travelers from and to the district at risk are completely suppressed. Note that this intervention is independent of the value of $\kappa$, which controls the overall mobility in the system even in the absence of restrictions. It is also important to remark that this is again a best-case scenario since in reality trips involving affected areas were allowed for a variety of circumstances.
2. Transmissibility reduction. Localized lockdowns usually come with additional measures to try to control the spreading within the affected area. This may include a rise in disease awareness through mass media and authorities’ advertisements and actions like occupancy limits and mandatory wearing of face masks. The combination of such actions would translate into a reduction of the transmissibility, its magnitude being dependent on people’s compliance and the intensity and effectiveness of those same measures. To model this, we set a local transmissibility rate for each closed district *i*, $\beta _i=\chi \beta$, where $\beta$ is the general transmissibility rate related to the basic reproductive number of the disease $R_0=\beta T_I$, and $\chi$ is the fraction of the reduction in transmissibility. For normal districts, $\chi =1$, but when a district is under lockdown, this quantity is $\chi <1$.

Once these measures are in place for a district at risk, the restriction remains until the outbreak ends in the whole system. That is, when the global incidence is $I=0$. In reality, however, once the affected areas stopped fulfilling the lockdown requirements, these restrictions were lifted. Since we are not seeking a replication of the Madrid epidemiological trajectory, but a general exploration and understanding of the basic dynamics under these types of lockdowns, we limit ourselves to studying what happens in this extreme scenario.

### Population and mobility data

The metapopulation model is informed by real population and mobility data from Madrid. Madrid is the capital city of Spain with a population of $N=3,312,310$ inhabitants in 2020. The prime administrative divisions of the city are $V=21$ *distritos* (districts). The model does not resolve at the basic health zone (BHZ) level, which is the one that was actually considered for the PLs, due to the lack of more fine-grained mobility data. In Table 3, we collect the population size at the district level $N_i$ (note that a BHZ generally encompasses between 5000 and 25,000 inhabitants). Regarding mobility data, we obtained interdistrict mobility flows provided by the Spanish Ministry of Transport as part of a survey aimed at analyzing changes in mobility patterns within Spain amid the COVID-19 crisis and at evaluating the effect of mobility restrictions^41^. This survey is based on cell phone data provided by cell phone carriers.

The data of interest spans a period ranging from March 2020 until May 2021. There is also a week of data in February 2020, from day 14 to day 21, used as a baseline reference for comparisons with the COVID-19 period. We refer to this period as the regular mobility scenario. We took only this reference period since we want to inform our urban model of Madrid with regular or unperturbed data. These data are used to build the origin-destination matrix ${\textbf {M}}$. To do so, we average the mobility flows between *i* and *j* for the whole week so that ${\textbf {M}}$ is time-independent.

**Table 3:** Population data (2020) of the 21 districts in which Madrid is subdivided.

District	$N_i$	District	$N_i$	District	$N_i$
Centro	141,236	Fuencarral	247,692	Ciudad Lineal	216,818
Arganzuela	154,243	Moncloa	120,834	Hortaleza	193,228
Retiro	118,557	Latina	240,155	Villaverde	154,808
Salamanca	146,016	Carabanchel	258,633	Villa de Vallecas	114,733
Chamartín	145,700	Usera	142,454	Vicálvaro	75,485
Tetuán	159,849	Puente de Vallecas	239,057	San Blas	160,258
Chamberí	138,667	Moratalaz	93,810	Barajas	50,077

## Data Availability

The epidemiological data is publicly available at^35^. The raw mobility data is publicly available at^41^. The code with the model used for the simulations, together with an example script and plot, and the curated mobility data for the city of Madrid used for informing the model can be accessed at https://github.com/phononautomata/madrid.
